# The impact of transcranial random noise stimulation (tRNS) on alpha coherence and verbal divergent thinking

**DOI:** 10.1162/netn_a_00446

**Published:** 2025-04-30

**Authors:** Magdalena Camenzind, Rahel A. Steuri, Branislav Savic, Fred W. Mast, René M. Müri, Aleksandra K. Eberhard-Moscicka

**Affiliations:** Perception and Eye Movement Laboratory, Departments of Neurology and BioMedical Research, University Hospital Inselspital, University of Bern, Bern, Switzerland; Graduate School for Health Sciences (GHS); Department of Psychology, University of Bern, Bern, Switzerland; Department of Neurology, Inselspital, Bern University Hospital, University of Bern, Switzerland; Gerontechnology and Rehabilitation Group, ARTORG Center for Biomedical Engineering Research, University of Bern, Bern, Switzerland

**Keywords:** Noninvasive brain stimulation (NIBS), Weighted node degree (wND), Large scale networks, Neural modulation, Verbal creativity, Dual process model of creativity

## Abstract

Random noise stimulation (tRNS) applied to the dorsolateral prefrontal cortex (DLPFC) enhances fluency and originality in verbal divergent thinking tasks. However, the underlying neural mechanisms of this behavioral change remain unclear. Given that the DLPFC is a key node of the executive control network (ECN) and that creativity is a two-stage process in which the ECN is primarily involved in the final idea selection stage, application of tRNS to this region shall not only result in an increase of originality and flexibility but also in a modulation of EEG activity. To test these assumptions, we collected 256-channel EEG of 40 participants before and after tRNS/sham applied to the DLPFC, during which participants performed two verbal creativity tasks. To assess stimulation-induced connectivity changes and to capture large-scale cortical communication, a source space alpha (8–12 Hz) imaginary coherence was calculated. We found that the tRNS-induced improvements in originality and flexibility were associated with bilateral DLPFC alpha coherence changes. From a large-scale networks perspective, these results suggest that tRNS-induced ECN activity is associated with increased originality and flexibility, potentially by enhancing selectivity in the idea evaluation phase. This study, for the first time, indicates a link between neurophysiological activity and tRNS-induced changes in verbal creativity.

## Author Summary

This study investigates the impact of transcranial random noise stimulation (tRNS) on alpha coherence and the link between neurophysiological and tRNS-induced effects on creativity. Given the dorsolateral prefrontal cortex’s (DLPFC) role in creativity’s final idea selection stage, 40 participants underwent 256-channel EEG recordings immediately before and after tRNS applied to the DLPFC. Using a within-subject design with active and sham tRNS sessions, we found that the tRNS-induced changes in originality and flexibility were linked to changes in alpha coherence in both left and right DLPFCs, analyzed in source space. These results suggest a network-level impact of tRNS on creativity, potentially enhancing executive control network activity for improved idea evaluation and indicate that the effect of tRNS is explained by task-related brain activity.

## INTRODUCTION

### Concepts and Assessment of Creativity

Creativity as a higher order cognitive process is a product of complex and interconnected neural networks engaging the entire brain (Dietrich & Kanso, 2010). Various attempts have been made to operationalize this complex phenomenon, most of which focus on divergent thinking (DT) and convergent thinking (CT), that is, two measurable cognitive components of creative cognition (Guilford, 1967). DT is typically required when there is more than one correct solution, vague selection criteria are given, or increased idea generation is desired. “Flexibility of the mind” is associated with the DT style (Zhang et al., 2014), and the alternative uses task (AUT) is classically used for its assessment in the verbal domain (Guilford, 1967). Additionally, given that half of the variance in DT ability can be attributed to associative abilities (Benedek et al., 2012), associative processes are central to DT. This link underscores the relevance of the associative fluency task (AFT) as a measure for approximating DT performance in creativity research (Camenzind et al., 2024; Diana et al., 2021; Wang et al., 2024). Conversely, CT requires persistence and focus to find one single solution of well-defined problems (Guilford, 1950; Runco, 2010; W. Zhang et al., 2020). Creativity can be further described by the dual process model (Basadur et al., 1982; Weinberger et al., 2017), which entails idea generation that is followed by idea evaluation and consequently selection (Lloyd-Cox et al., 2022). The link between creativity as a two-stage process and the concepts of DT and CT becomes apparent in that the idea generation stage is based on DT, while the idea evaluation and selection stage are based on CT. The “Dual Pathway to Creativity Model” (Nijstad et al., 2010) also includes the concepts of flexibility and persistence. The latter is primarily involved in CT, whereas the former is predominantly involved in DT.

### Large-Scale Networks

Given the multifaceted nature of creativity, it is not surprising that the generation of original ideas requires functional interaction between several brain regions (Benedek, 2018; Benedek & Fink, 2019; Dietrich, 2004; Weinberger et al., 2017) or even large-scale brain networks (Beaty et al., 2016, 2019; Shi et al., 2018). Multiple fMRI studies highlighted the importance of the coupling between the executive control network (ECN) and the default mode network (DMN) in creativity research (Beaty et al., 2015, 2016, 2018). Whereas the DMN is composed of the posterior cingulate cortex (PCC) and medial prefrontal areas with further prominent nodes in the medial temporal lobe and angular gyrus (Menon, 2015; Raichle et al., 2001), the ECN is a frontoparietal system mainly constituted of the dorsolateral prefrontal cortex (DLPFC) and the lateral posterior parietal cortex (PPC). Another large-scale network is formed by the salience network (SN), principally involving the anterior cingulate cortex (ACC) and the frontal insular cortex (Menon, 2011). The DMN is active when participants are not focused on the outside world but turn their attention inward to extract new information from semantic and episodic memories through the temporal lobe subsystem, thus learning from past experiences (Menon, 2011). While the frontoparietal ECN evaluates and selects information according to specific task goals, the SN is responsible for flexible switching between the DMN and the frontoparietal ECN (Li He et al., 2019).

### Alpha Coherence

Next to fMRI and other neuroimaging methods measuring brain activity at a given moment (e.g., positron emission tomography [PET] or local field potential recordings), communication between nodes of large-scale networks can be measured by EEG (Bressler & Menon, 2010). Most prominently, alpha coherence weighted node degree (wND) is employed as a proxy of functional coupling between different brain areas (Guggisberg et al., 2008) and large-scale cortical communication (Chapeton et al., 2019). Specifically, alpha band functional connectivity (i.e., the absolute imaginary part of coherence; Nolte et al., 2004) between a given brain area and the rest of the brain correlates with the performance in a task relying on this area (e.g., an increase in alpha-band coherence between the hand motor cortex and the rest of the brain was associated with an improvement of motor function; Mottaz et al., 2015).

Multiple EEG studies reported creativity-associated functional coupling patterns (e.g., Fink, Rominger, et al., 2018). In a creativity task consisting of a divergent stage (i.e., idea generation) and a convergent stage (i.e., idea selection), an increased frontotemporal alpha coherence was observed for the generation of novel as compared with regular solutions, which may reflect the selective inhibition of irrelevant semantic information (Zhou et al., 2018). Importantly, the frontotemporal alpha coherence was increased at the beginning of the creative process, possibly indicating a transition from divergent to CT (Zhou et al., 2018). These results align with the described increased coupling of the DMN (e.g., medial temporal cortex) and the ECN (e.g., DLPFC) toward the evaluative stage of the creative process (Beaty et al., 2015, 2016). Adding to this evidence, while an early increase in functional communication between frontal and parietal/occipital areas within the ideation process in the AUT was observed for more original individuals, this long-distance functional communication was delayed in less original individuals (Rominger et al., 2019). This finding was interpreted as an early activation of top-down executive control processes regulating sensory and visual information and higher internal attention. The increase in functional coupling (alpha-coherence) during the idea generation phase was confirmed for the frontoparietal and occipital network (Rominger et al., 2020), whereas the frontocentral as well as the frontotemporal networks increased their functional coupling during the transition from the idea generation to the elaboration phase (Rominger et al., 2022). This increased functional coupling with frontal brain regions was hypothesized to reflect enhanced executive processes relatively early in the creative process, supporting the emergence of highly original thoughts (Rominger et al., 2022). Concurring with these findings, an increased functional coupling in the alpha band was observed between frontal and parietal/temporal regions during AUT performance (Fu et al., 2022).

While other functional connectivity measures (e.g., phase amplitude coupling) and frequency bands (e.g., theta, beta) are also relevant, alpha coherence is particularly suitable for studying creativity due to its role in cortical inhibition (Jensen & Mazaheri, 2010; Mathewson et al., 2011) and large-scale network communication (Beaty et al., 2015, 2016), crucial for selective attention and cognitive control during the creative process. These are particularly important in creative thinking, where the ability to manage competing thoughts and filter out distractions supports idea generation and evaluation (Zhou et al., 2018). This consistent link between alpha coherence and creative cognition supports the choice of alpha coherence as a proxy for functional coupling (Fink, Rominger, et al., 2018; Fink & Benedek, 2014; Fu et al., 2022; Rominger et al., 2022).

### Transcranial Electrical Stimulation (tES) and Creativity

tES is a noninvasive brain stimulation technique that uses a weak current passing through the skull to modulate brain activity. tES, applied as direct current (i.e., transcranial direct current stimulation [tDCS]) may enhance performance in tasks requiring fluency (Chrysikou et al., 2021) and idea selection (Weinberger et al., 2017). Various studies examined the effects of tDCS on creativity-related brain networks, revealing distinct changes in functional connectivity and cognitive processes, for example, within the DMN (Koizumi et al., 2020), in DT and cognitive flexibility (Khalil et al., 2020), and exploring the differential impacts of cathodal and anodal stimulation on metaphor generation (Lifshitz-Ben-Basat & Mashal, 2021).

Also, tES applied in the form of alternating current (i.e., transcranial alternating current stimulation) that enables targeting specific brain oscillations, for example, alpha frequencies, enhanced figural creativity (Lustenberger et al., 2015), and increased ideational fluency in verbal tasks (Grabner et al., 2018).

Peña et al. (2023) compared transcranial random noise stimulation (tRNS) with tDCS, revealing that tRNS had a more pronounced effect on aspects of creativity such as fluency and originality compared with tDCS. This finding supports the efficacy of tRNS in enhancing creative tasks and provides a direct comparison for evaluating the effects of different brain stimulation methods (Peña, Sampedro, et al., 2022). tRNS uses alternating current of normally distributed random amplitudes and randomly selected frequencies (Antal & Herrmann, 2016; typically ranging between 100 and 700 Hz, Moret et al., 2019) to increase sensitivity of neurons to weak inputs (Pavan et al., 2019). Compared with tDCS, tRNS yields certain advantages such as higher cutaneous perception thresholds (Ambrus et al., 2010) and consequently better experimental control. Whereas stochastic resonance (i.e., enhanced detectability of weak stimuli by adding white noise) and repeated opening of Na+ channels were suggested as possible neurophysiological mechanisms underlying tRNS (Terney et al., 2008; van der Groen et al., 2022), the knowledge about its mechanism of action remains scarce (Terney et al., 2008).

In a study of verbal creativity, Peña et al. (2019) reported a tRNS-induced increase in fluency and originality in the AUT. In a follow-up study, Peña et al. (2020) reported an increase in figural originality following the application of tRNS to the PPC (compared with DLPFC as applied in Peña et al., 2019), which they attributed to the role of PPC in inward attention. Moreover, in a combined tDCS-tRNS protocol, with the anode over the left DLPFC and the cathode over the left inferior frontal gyrus (IFG), Peña et al. (2023) reported improved performance in CT (i.e., Remote Associates Task) and DT tasks (i.e., picture completion subtest of the Torrance Test of Creative Thinking; Torrance, 1966). These findings indicate a beneficial effect of the combined tDCS-tRNS paradigm on switching efficiency, that is, switching between persistence (relevant for CT) and flexibility (relevant for DT) as discussed in the context of the “Dual Pathway to Creativity Model” (Nijstad et al., 2010).

While the current findings suggest a beneficial effect of tRNS applied to DLPFC on verbal creativity at the level of behavior, the underlying neurophysiological mechanisms of this effect are largely unknown. Thus, the aims of the present study were: (a) to measure the effect of tRNS applied to the DLPFC on alpha coherence (on source level) in the whole brain and (b) to investigate whether the tRNS-induced change in alpha coherence between the frontal areas and the rest of the brain can be predicted by the tRNS-induced changes in behavioral performance in two verbal DT creativity tasks. To this end, 20-min tRNS and sham were applied to the bilateral DLPFCs of 40 participants, who concurrently performed the AUT and the AFT. Resting-state EEG was measured prior and after stimulation, and the change in the respective alpha coherence was calculated.

## METHODS

### Participants

Forty-two healthy native German speakers took part in the study, two of which had to be excluded due to corrosion of the tRNS electrodes (i.e., the corroded electrodes lead to high electrode-impedances that automatically terminated the tRNS stimulation shortly after stimulation onset). As such, the final sample consisted of 40 participants (3 left-handed; 25 females) with a mean age of 29 years (*SD* = 12.7). All participants had normal or corrected-to-normal visual acuity and gave their written informed consent prior to participation. The study was approved by the Ethics Committee of the University of Berne and was conducted in accordance with the code of ethics of the World Medical Association (Declaration of Helsinki).

### Procedure

**Figure 1.**
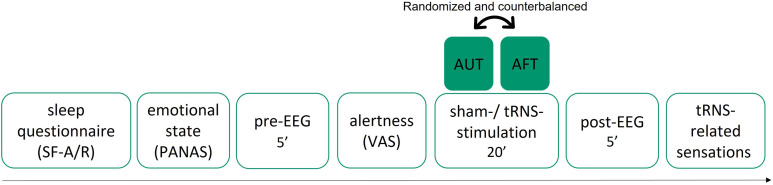
Schematic of the experimental sessions. The active tRNS and the sham tRNS sessions were identical except for the stimulation condition (i.e., active tRNS vs. sham tRNS), the order of which was randomized and counterbalanced across participants. Each session initiated with a sleep questionnaire, a questionnaire assessing affective state, and an alertness scale. Following the application of the tRNS electrodes and the EEG net, 5 min of resting-state EEG (i.e., pre-tRNS) were recorded. Subsequently, participants completed two verbal creative thinking tasks (i.e., AUT and AFT) during which either active or sham tRNS (task order randomized and counterbalanced across conditions) was applied. Immediately after the stimulation, another 5 min of resting-state EEG (i.e., post-tRNS) were recorded. The session concluded with a questionnaire on tRNS-related sensations.

The study procedure included three sessions conducted at the same time of the day (i.e., screening session and two experimental sessions: tRNS session and sham tRNS session; see Figure 1). The sessions took place at the Perception and Eye Movement Laboratory of the Inselspital Bern and were separated by 1-week intervals. During the screening session, basic demographic data and baseline measures of creativity, cognition, and personality were collected (for details on creativity, cognition, and personality measures, see Supporting Information S1.1–S1.6 and Supporting Information Table ST1). Except for the stimulation condition (i.e., active tRNS vs. sham tRNS, the order of which was randomized and counterbalanced across study participants), the experimental procedure of the tRNS session and the sham tRNS session was identical.

**Figure 2.**
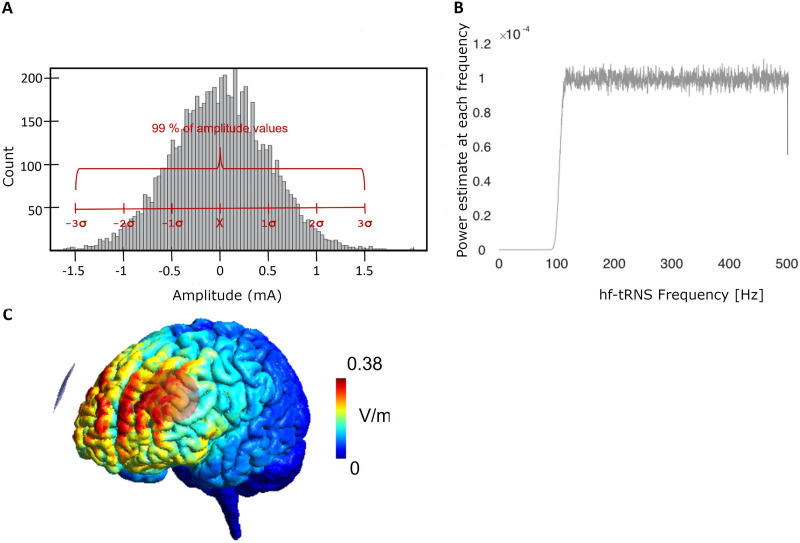
(A). Normal distribution of random noise in tRNS with zero offset and baseline-to-peak current intensity variation of 1.5 mA. The figure was adapted from van der Groen et al. (2022). (B) Power spectrum of transcranial tRNS signal. High-frequency tRNS was used (101–5000 Hz), whereby power was constant for all frequencies (white noise). The figure was adapted from Potok et al. (2022). (C) Simulation of the electrical field as induced by tRNS. Stimulation electrodes were placed on the left and right dorsolateral prefrontal cortices (DLPFC, i.e., F3 and F4 according to the International 10–20 system). The simulation was performed with SimNIBS (Saturnino et al., 2021; Version 3.2.1).

In all three sessions, data on sleep quality (Supporting Information S1.7), positive and negative affective state (Supporting Information S1.8), and alertness (Supporting Information S1.9) were collected (Supporting Information Table ST1). Both experimental sessions began with the application of two tRNS electrodes and the EEG net (see the Stimulation Protocol section below) after which 5 min of resting-state EEG were recorded (i.e., pre-tRNS EEG; see EEG recordings below). After disconnecting the EEG net from the EEG system, the portable tRNS device was attached to the tRNS electrodes, which were placed on the participant’s DLPFC bilaterally (see Figure 2C). All study participants received both active and sham tRNS during which they completed two verbal DT tasks (presented in a randomized and counterbalanced order between the conditions; see AUT and AFT below). Following the termination of the stimulation protocol (see the Stimulation Protocol section below), the tRNS device was removed and another 5 min of resting-state EEG were recorded (i.e., post-tRNS EEG). Each experimental session was concluded by a questionnaire on the tRNS-related sensations (Supporting Information Table ST2 and Figure 1).

All experimental tasks were programmed in Unity and administered on a Lenovo Yoga notebook 720-15IKB (15.6 Zoll, i7, 16GB RAM). Verbal responses were recorded by means of an external microphone (Jabra PHS001U) and were further transcribed by the study experimenter.

### AUT

In the AUT, participants were presented with six objects and their common use and were asked to generate alternative uses for each of the given objects (i.e., three words in each experimental session) within 3 min. The original English object names were translated to German (i.e., [safety pin] “Sicherheitsnadel,” [chair] “Stuhl,” [watch] “Uhr,” [automobile tire] “Autoreifen,” [eyeglasses] “Brille,” and [wooden pencil] “Holzbleistift”; Guilford et al., 1978) and sorted into two parallel sets that were presented in a random order during Sessions 2 and 3. The two sets were counterbalanced between the two conditions. The object names (font type: Arial and font size: 40, bold) were presented in the center of the Lenovo Yoga notebook screen with their common use in parentheses below them. Participants’ answers were evaluated on fluency, flexibility, and originality. Fluency score was based on the total number of valid answers according to Guilford et al. (1978). Flexibility score was based on a machine-learning approach that uses German word-vectors to quantify semantic distances between the answers (Camenzind et al., 2024). Specifically, a word vector model was trained on a diverse 0.67-billion-word German text corpus and synonymous words were linked and assigned the same vector to account for a potential overestimation of flexibility. This method provides an objective measure of flexibility in the German language, complementing traditional fluency and originality scores in DT research and offering a more time- and cost-effective assessment of creativity performance. Originality score was based on the statistical infrequency of a given alternative use within the entire pool of the alternative uses provided by all the participants (i.e., the frequency of each answer across the answers provided by all participants was evaluated and inverted to facilitate interpretation). The mean fluency, flexibility, and originality values across all answers of each participant were calculated per object and were further averaged over the three objects, resulting in the final fluency, flexibility, and originality score per participant per session.

### AFT

In the AFT (Benedek et al., 2012), participants were presented with a total of six target words (i.e., three words in each experimental session) and they had 3 min per word to name everything that came to their mind when thinking of that. The six target words (derived from the German translation of the Kent-Rosanoff Word Association Test [Russell, 1970], i.e., “Teppich” [carpet], “Schaf” [sheep], “Tisch” [table], “Adler” [eagle], “Haus” [house], and “Schere” [scissor]) were sorted into two parallel sets that were presented in a randomized order on the Lenovo Yoga notebook screen (font type: Arial and font size: 40, bold). The two sets were counterbalanced between the two conditions. All answers were analyzed on fluency, flexibility, and originality. Fluency score was based on the number of answers per target word (with double listings excluded). Flexibility and originality scores were calculated analogous to the corresponding AUT scores (see the AUT section). Similarly to the AUT, the mean fluency, flexibility, and originality values across all answers of each participant were calculated per object and were further averaged over the three objects, resulting in the final fluency, flexibility, and originality score per participant per session.

### Stimulation Protocol

We employed the tRNS protocol by Peña et al. (2019) who reported a positive effect of tRNS on verbal DT. In brief, study participants received alternating current with a baseline-to-peak intensity of 1.5 mA (Figure 2A) and random frequencies between 100 and 500 Hz (Figure 2B) bilaterally to their DLPFCs. Two circular electrodes (2.5 cm in diameter) were attached to the scalp with conductive paste (Ten20 conductive paste, Weaver and Company, USA) at the positions corresponding to the location of the DLPFC on the scalp (i.e., F3 and F4 according to the International 10–20 System). Importantly, these standard positions do not precisely map to specific anatomical regions and the individual anatomical variability was not considered in this study; instead, a template-based approach was used (see the Connectivity Analysis section). Therefore, the stimulation is likely to affect a broader area of the frontal lobe rather than targeting specific anatomical regions. The thickness of the layer of the conductive paste was standardized to 0.5 cm by using a custom-made mold, and it was introduced as a parameter when simulating the electric field resulting from the tRNS by means of SimNIBS (Saturnino et al., 2021; Version 3.2.1; Figure 2C). For the stimulation, a portable, battery-driven stimulation device was used (Neuroelectrics Inc., Barcelona), which was connected to a laptop computer via Bluetooth. Prior and during tRNS application, the impedances of both electrodes were maintained below 10 kΩ. In the active condition, electrical current was delivered continuously for 20 min, with a ramp-up and ramp-down of 30 sec each. While the duration of the sham condition was identical to the active tRNS condition (i.e., 20 min), it only consisted of an initial 30-sec ramp-up that was immediately followed by a 30-sec ramp down with no subsequent electrical stimulation.

### EEG Recordings

A resting-state 256-channel EEG (low-profile MicroCel Geodesic Sensor Net; EGI NA 400 amplifier, Magstim EGI, USA) was recorded against the Cz reference, at a sampling rate of 1000 Hz. Cz was placed at the 50% point of the distances between the nasion and inion as well as between the peri-auricular points, and the EEG electrodes were filled with an electrolyte gel (electro-gel, Electro-Cap International). As modern high-input impedance amplifiers and their accurate digital filters for power noise provide excellent EEG signal collection even at higher electrode impedances (Ferree et al., 2001), the electrode impedance was kept below 50 kΩ (cf. e.g., Eberhard-Moscicka et al., 2021; Franklin et al., 2007; Rihs et al., 2007). During the post- and pre-tRNS EEG recordings, participants sat in a comfortable chair, opening and closing their eyes alternately every 35 sec as instructed by the experimenter. Whenever they had their eyes open, participants were asked to fixate on a cross drawn on the wall in front of them.

### Data Preprocessing

Data preprocessing was implemented in custom-written scripts and functions that were run in MATLAB (Version R2022a), using EEGLab (Version 2022) and CSC toolbox (Mensen et al., 2016). The preprocessing steps included manual detection of bad channels and identification of bad stretches that were further removed from the continuous data. Physiological artifacts such as eye blinks, saccades, heartbeat, or muscle were corrected by means of an independent component analysis (Adaptive Mixture of Independent Component Analysis plug-in for EEG Lab; Leutheuser et al., 2013). The corrected files were digitally high-pass (0.5 Hz) and low-pass (40 Hz) filtered, and the previously removed channels were interpolated using spline interpolation (Perrin et al., 1987). Signals were then referenced to the average activity over all channels, and the recording Cz-electrode was used as an additional electrode for further data processing. Interpolation was performed prior to average referencing to avoid any potential lateralization bias from the artifactual channels and to keep the degrees of freedom for channel-level calculations consistent at the group level. In the final step, the continuous EEG recording was segmented into “eyes open” and “eyes closed” epochs. To avoid effects of alpha desynchronization due to visual input, the subsequent statistical analyses were conducted on the eyes-closed data (Barry et al., 2007; Kan et al., 2017; Niedermeyer & da Silva, 2005; Schomer & Da Silva, 2012; mean duration of eyes closed reported in minutes and standard deviations in parentheses: for the pre-tRNS: 1.94 [0.25], post-tRNS: 1.98 [0.17], presham: 1.97 [0.29], and postsham: 1.96 [0.35]).

### Connectivity Analysis

Connectivity analysis was performed with Brainstorm (Tadel et al., 2011), a documented and freely available software under the open-source General Public License (https://neuroimage.usc.edu/brainstorm) and custom-written MATLAB and Python scripts (available at the URL https://osf.io/r67fj/). In a first step, brain activity accounting for the scalp recordings had to be estimated. Estimating brain activity at multiple locations from fewer sensors is commonly referred to as the ill-posed inverse problem (Pascual-Marqui, 1999). Given that in the present study the participants’ individual anatomies were not available, the Brainstorm default anatomy was used, and the Boundary Element Method (Gramfort et al., 2010; Kybic et al., 2005) was applied to calculate the volume conduction model of the participants’ head. This study used the “minimum-norm imaging” (i.e., MN imaging) source estimation approach as featured by Brainstorm. The MN imaging estimates the amplitude of brain sources distributed across the brain that are constrained to the cortex. It requires specification of noise statistics via the so-called noise covariance matrix. For this study, the noise covariance was assumed to be uniform across all sensors. Final sources were estimated in terms of best fit to sensor data and lowest overall amplitude of brain activity. Moreover, due to the inhomogeneous sensitivity of EEG to depth and orientation of current flow, it is recommended to standardize the current density map (Dale et al., 2000). In the present study, this was achieved by means of the dynamical Statistical Parametric Mapping (Dale et al., 2000), where the current density maps were scaled with respect to the noise covariance and resulted in *z*-scores. Given the use of the MRI template instead of the individual anatomies, a loose orientation model with three dipoles per source grid location (vertex), instead of the standard one main dipole, was calculated to account for anatomical and physiological uncertainties. The amplitude of the two additional dipoles was limited to a fraction (0.2) of the main dipole. The size of the resulting kernel was 3*NbVertices*NbChannels. In the current study, stimulation sites were based on the 10–20 system (i.e., F3 and F4); hence, they do not precisely map to specific anatomical regions. Moreover, the stimulation likely affects a broader area of the frontal lobe, and individual anatomical variability was not considered, as a template-based approach was used. Thus, anatomical terms used here are general indicators based on source analysis and should be considered putative.

The magnitude square coherence is a measure of covariance between two signals in a given frequency domain. Notably, this measure cannot distinguish between areas that have a linear relationship/coherent activity and areas that originate from the same source. In the latter case, the measured activity can be wrongly considered as a coherent activity of two areas (volume conduction problem). In the current study, this problem was addressed by implementing the measure of imaginary coherence (Nolte et al., 2004) allowing the assumption that all nonzero coherence values are not due to volume conduction:

ICxyf=ImSxyfSxxfSyyf

Where *IC*_*xy*_(*f*) represents the imaginary coherence value at frequency *f* for areas *x* and *y* of the brain, *S*_*xx*_ represents the power spectrum of area *x*, and *S*_*xy*_ represents the cross-power spectrum of areas *x* and *y*, resulting in IC values ranging from 0 to 1 and corresponding to the covariance of two signals in the given frequency domain (with high IC values indicating high information transmission between the given brain regions, and low IC values indicating low information transmission). To calculate the IC, the duration for the spectrum estimation was set to 1 s, and the percentage of overlap between consecutive time windows was set to 50%. Alpha frequencies were considered with the highest frequency of interest of 12 Hz (i.e., 8–12 Hz). The calculations were not performed for every vertex; instead, scouts (i.e., regions of interest) were defined. The IC value for each scout consisted of the mean IC values of all the vertices within the scouts. For the definition of scouts, the anatomical Mindboggle Atlas (Klein et al., 2017), as implemented in FreeSurfer (https://freesurfer.net/fswiki/CorticalParcellation) and available in Brainstorm, was used. Coherence calculations were further performed for the 62 scouts as defined by the Mindboggle Atlas (Klein et al., 2017). In the final step, the wND was calculated (i.e., the sum of the scouts’ ICs with all the other scouts; Manuel et al., 2018; Newman, 2004; Nicolo et al., 2015), whereby the wND describes the overall importance of a scout within the brain network (Stam & van Straaten, 2012). Furthermore, wNDs were normalized by subtracting the mean wND of all scouts from the values at the target scouts and by dividing them by the standard deviation of the wND values over all the scouts (i.e., *z*-scores were calculated; Dubovik et al., 2012).

Although tRNS was applied to the DLPFC (i.e., F3 and F4 electrode placement according to the International 10–20 System), as depicted by the simulation performed with SimNibs (Saturnino et al., 2015), the entire frontal cortex was stimulated (see Figure 2C). As such, the *z*-transformed wNDs of the left and right hemispheric frontal areas were averaged to form a wND value for the left and right frontal areas. Subsequently, the left and right wNDs of the pre-tRNS EEG recording were subtracted from the corresponding values of the post-tRNS EEG recording and resulted in wND change values for the left and right frontal areas, which were calculated per participant and per experimental condition (i.e., tRNS and sham).

### Statistical Analysis

To address the first aim, we performed an exploratory analysis examining the effect of tRNS on imaginary coherence between the 62 scouts as defined by the anatomical Mindboggle Atlas (Klein et al., 2017). To this end, nonparametric statistical testing was performed on the raw IC change data (i.e., pre-tRNS IC subtracted from post-tRNS IC) and compared with IC changes between the different scouts across the two conditions (i.e., tRNS and sham). In the present study, permutation-based, nonparametric student’s *t*-test statistic for paired samples and the Monte Carlo method (Maris & Oostenveld, 2007; 5,000 draws from the permutation distribution) were used, and the resulting *p* values were corrected for multiple comparisons using the false discovery rate method. Prior to this exploratory analysis, two paired *t* tests (i.e., tRNS-pre vs. sham-pre for the left and the right DLPFCs) were conducted to control for possible confounds arising from baseline differences in alpha coherence between the two experimental sessions (i.e., tRNS and sham).

To address the second aim, for each participant, the difference of frontal wND change scores (i.e., post-pre) were calculated between the two stimulation conditions (i.e., tRNS vs. sham). In the following, the change of the wND change value between the two stimulation conditions is referred to as wND change. Also, for the three measures (i.e., fluency, flexibility, and originality) within the two tasks (i.e., AUT and AFT), the differences between the two stimulation conditions (i.e., tRNS-sham) were calculated for all participants. In line with interventional studies, which aim to manipulate behavior (here, tRNS applied to the DLPFC during AUT and AFT) to infer about changes in neural activity; four multiple regressions were run to predict whether changes in task performance (i.e., AUT and AFT) contributed to the explained variance in the functional connectivity (i.e., wND change values in the left and right frontal areas).

The analyses of the behavioral performance of both tasks (i.e., AUT and AFT) and the analyses of the post-pre tRNS and sham alpha coherence changes are reported in the Supporting Information S2.1 and S2.2.

## RESULTS

### tRNS Modulates Alpha Coherence

The first aim of this study was to explore the effect of tRNS on overall brain coherence. Paired *t* tests confirmed the absence of baseline differences in alpha coherence between the experimental conditions (i.e., tRNS vs. sham; right DLPFC: *t*(39) = −0.827, *p* = 0.413; left DLPFC: *t*(39) = 1.206, *p* = 0.235). While the paired *t* tests of the post-pre alpha coherence changes between the tRNS and sham conditions in the left and right frontal areas revealed no significant effects (see Supporting Information S2.1, SF1), permutation-based, nonparametric *t* tests for paired samples (reported below) revealed noteworthy changes in alpha coherence.

**Figure 3.**
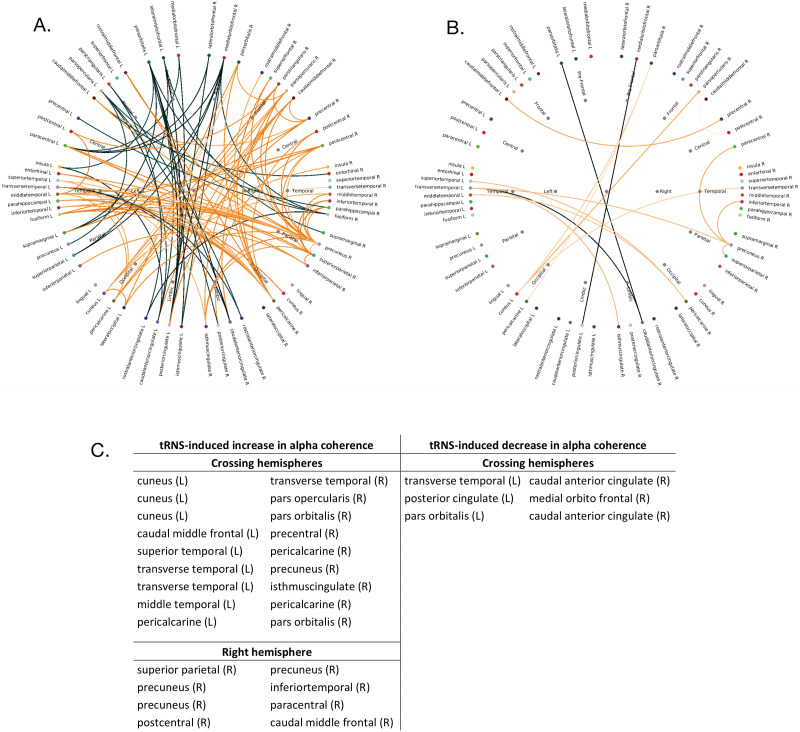
Permutation-based, nonparametric *t* tests for paired samples were calculated on raw coherence data. Significant (A, *p* < 0.05 and B, *p* < 0.01) tRNS-induced changes in imaginary coherence (IC) difference values (i.e., post-pre) between the brain areas (as defined by the anatomical Mindboggle Atlas; Klein et al., 2017) are represented by the black and orange lines. Black lines indicate negative *t* values (i.e., decreased alpha coherence following tRNS application), and orange lines indicate positive *t* values (i.e., increased alpha coherence following tRNS application). (C) Tabular summary of the positive (left) and negative (right) coherence changes between the nodes from Panel B. L – left hemisphere, R – right hemisphere.

In the connectivity graph (Figure 3A and B), the black and orange lines indicate significant changes in the IC change values (i.e., post-tRNS IC – pre-tRNS IC) between the brain areas as defined by the anatomical Mindboggle Atlas (Klein et al., 2017). The difference of the IC change values was calculated by subtracting the IC change values of the sham condition from the IC change values of the tRNS condition. Whereas the black lines indicate a negative change (i.e., negative *t* values, indicating a poststimulation decrease in IC coherence between the given scouts), the orange lines indicate a positive change (i.e., positive *t* values, indicating a poststimulation increase in IC coherence; see Figure 3A and B). Compared with Panel A, where the *p* values are set below 0.05, in Panel B the threshold is more rigid (i.e., *p* < 0.01; see Figure 3A and B). As stated in the Connectivity Analysis section, anatomical terms used in this study are general indicators based on source analysis rather than precise anatomical designations. Thus, all regions discussed below should be considered putative.

Permutation-based, nonparametric *t* tests for paired samples (*p* < 0.01) indicated a post-tRNS increase in the coherence between the left occipital areas and the right frontal, right prefrontal, and right temporal areas; and between the left temporal and the right parietal, occipital, and the limbic regions. Additionally, a post-tRNS increase in coherence was found between the left frontal and the right central areas. At the same time, decreased coherence between the left limbic and the right prefrontal regions as well as between the right limbic and the left prefrontal regions was observed.

### The Impact of tRNS on Verbal Creativity Performance

**Figure 4.**
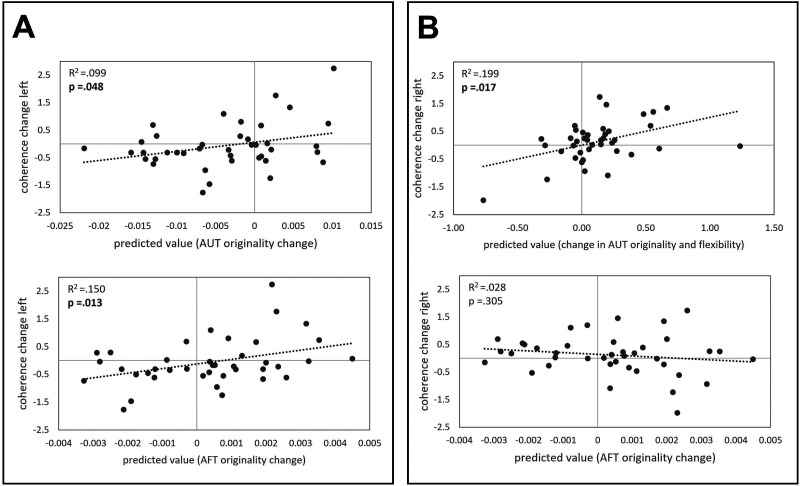
Scatterplots of the left (A) and the right hemispheric (B) tRNS-induced average change of wND of the frontal cortex (*y* axis) and tRNS-induced change in the AUT and the AFT performance (*x* axis). Significant *p* values are marked in bold.

Analysis of the task performance is reported in the Supporting Information (S2.2 and SF2). To address the second aim, that is, to explore whether the variance of the tRNS-induced wND change values within the left and right frontal areas can be predicted by the tRNS-induced change in the performance in the two verbal creativity tasks, multiple regressions were run separately for the AUT and the AFT. When entering all the AUT measures (i.e., fluency, originality, and flexibility) in one block using a backward regression method, originality alone explained about 10% of the variance in the left hemispheric tRNS-induced frontal wND change (*R*^2^ = 0.099, *p* = 0.048; Table 1 and Figure 4, upper part of Panel A), while originality and flexibility together explained almost 20% of the variance in the right hemispheric, tRNS-induced frontal wND change (*R*^2^ = 0.199, *p* = 0.017; Table 1 and Figure 4, upper part of Panel B). While originality indicated a negative association with the right-frontal alpha coherence (*β* = −23.349), flexibility indicated a positive association (*β* = 7.294; Table 1). To better understand these effects, two follow-up simple linear regressions were calculated, which revealed a marginal negative effect of originality (*β* = −0.187, *R*^2^ = 0.066), and a nonsignificant effect of flexibility (*β* = 0.265, *R*^2^ = 0.134) on the explained variance in the right hemispheric, tRNS-induced frontal wND change, respectively.

**Table 1.** Results of the final step of the multiple regression models using the backward stepwise procedure

	wND change frontal left			wND change frontal right		
	Task	Measures	*B*	*SE B*	*β*	*B*	*SE B*	*β*
Final step	AUT	Constant	0.063	0.141		0.043	0.117	
		Fluency	−0.035	0.061	−0.091^2^	−0.052	0.051	−0.156^2^
		Originality	**33.095**	**16.224**	**0.314** ^**1**^	−**23.349**	**13.487**	−0**.255**^**1**^
		Flexibility	0.860	3.712	0.037^2^	**7.294**	**2.951**	**0.364** ^**1**^
	AFT	Constant	−0.123	0.125		0.145	0.117	
		Fluency	−0.005	0.020	−0.037^2^	0.0210	0.018	0.193^2^
		Originality	**166.457**	**64.224**	**0.388** ^**1**^	−62.125	59.760	−0.166^2^
		Flexibility	3.780	5.480	0.107^2^	−2.301	5.110	−0.075^2^

Variables entered at the initial step as predictors of either left or right hemispheric tRNS-induced frontal wND change were the AUT and the AFT performance measures (i.e., fluency, originality, and flexibility). Variables that significantly predicted the wND changes are highlighted in **bold**. The regression model for the right frontal area was not significant for any of the three AFT predictors (*p* > 0.305).

^1^*p* < 0.05.

^2^*p* > 0.05.

In the multiple linear regression approach (i.e., backward regression method) applied for the AFT measures (i.e., fluency, originality, and flexibility), originality explained 15% of the variance in the wND change within the left frontal region (*R*^2^ = 0.15, *p* = 0.013), but none of the AFT measures contributed to the explained variance in the wND change within the right frontal region (*R*^2^ = 0.028, *p* = 0.305; Figure 4, lower part of Panels A and B).

## DISCUSSION

The present study examined the effect of tRNS on alpha coherence and the influence of tRNS on the performance in two DT verbal creativity tasks. To this end, we calculated the tRNS-induced changes in alpha coherence between different brain regions (as defined by the anatomical Mindboggle Atlas; Klein et al., 2017) and applied imaginary coherence, a measure of connectivity and functionality avoiding overestimation of connectivity due to sensor crosstalk and volume conduction. The measured activation changes between different brain regions, possibly indicating increased memory-based idea generation, mental imagery, and decreased activity within the DMN, as well as the impact of the DLPFC-tRNS on verbal DT, are discussed in the context of large-scale network communication. However, before discussing the findings in more detail, it shall be noted that the anatomical terms used in this study are intended as general indicators based on source analysis rather than precise anatomical designations. Hence, all regions discussed below should be considered putative.

### Increased Temporal–and Frontal–Parietal/Occipital Connectivity

The increased coupling between left hemispheric temporal regions and right hemispheric parietal/occipital regions (Figure 3A, B, and C) can be due to semantic processing (Li He et al., 2019), episodic memory retrieval (Cabeza et al., 2008; Wagner et al., 2005), and mental simulation of future self-referential situations (Schacter et al., 2007, 2012). Also, precuneus, cuneus, and visual cortices were found to be involved in mental imagery (Andreasen & Ramchandran, 2012; Cavanna & Trimble, 2006; Kosslyn et al., 2001; Kulakova et al., 2013). Indeed, our data indicated an increased coherence of the right precuneus and pericalcarine cortex with left temporal areas as well as coupling of the left cuneus and the pericalcarine cortex with the right frontal areas, possibly reflecting increased mental imagery. Overall, the observed connectivity changes between the frontal, posterior, and temporal regions may represent semantic processing, retrieval of long-term memories, and mental simulations that are important for idea generation during the creative process (Beaty et al., 2015; Ellamil et al., 2012). During the idea generation stage, semantic knowledge is drawn upon, and relevant memories are retrieved to mentally simulate future scenarios based on past experiences (Buckner et al., 2008; Schacter et al., 2007, 2012). These cognitive processes are essential for creativity since they facilitate exploration of alternative perspectives, integration of disparate information, and generation of innovative solutions to problems. By fostering interaction between language, memory, and imagination networks in the brain, the increased connectivity between the frontal, posterior, and temporal regions as found in our study may support fluidity and flexibility of thinking that are necessary for creative idea generation.

Furthermore, the increased tRNS-induced alpha coherence change between right frontal and left occipital areas, as reported here, can be explained by cognitive processes requiring internally oriented attention, for example, working memory that is primarily involved in idea evaluation and selection phases (Benedek, 2018; Fink, Pechthold, and Rominger, 2018; Fink & Benedek, 2014; Fu et al., 2022; Rominger et al., 2019). Concurring with our findings, an fMRI connectivity study described the time-course of functional coupling between frontal and parietal areas during AUT that was increased in the final stages of the creative process (Beaty et al., 2015).

### Large-Scale Networks in Creativity

Current creativity research supports the notion of creativity as a dual process (Basadur et al., 1982; Lloyd-Cox et al., 2022; Weinberger et al., 2017) that is further substantiated by the involvement of large-scale networks (Beaty et al., 2016, 2019; Shi et al., 2018). While idea generation is predominantly associated with a widespread activity of the DMN regions, idea evaluation is associated with enhanced coupling of DMN and ECN (Beaty et al., 2015, 2016). The DMN was proposed a task-negative network that is incompatible with performance of attention-intensive tasks (Weber et al., 2022) and that is active when individuals direct their attention inward (e.g., during the idea generation stage of the creative process; Buckner et al., 2008). Whereas the medial temporal lobe subsystem, as part of the DMN, provides memories of past experiences that help create self-relevant mental simulations and facilitate future planning (Buckner et al., 2008), the PCC as a further component of the DMN is involved in the retrieval of autobiographical memories and continuous memory processing (Weber et al., 2022).

Fox et al. (2018) emphasized the importance of intracranial recordings and stimulation targeting the DMN in advancing our understanding of its role in human behavior and cognition. Similarly, by employing direct cortical stimulation, Bartoli et al. (2023) demonstrated that creativity and mind-wandering engage the DMN with different temporal dynamics. This study provides causal evidence of the DMN’s involvement in creative thinking. Interestingly, stimulation of the DMN reduced creativity, particularly the originality of alternate uses in creative tasks (Bartoli et al., 2023) aligning with our finding of a reduced originality performance in the AUT. Furthermore, slow-wave intra-DMN synchronization during cognitive tasks suggests that frequency-specific changes in DMN connectivity are linked to cognitive processes (Das et al., 2022). Thus, changes in behavioral outcomes, for example, reduced originality, may reflect altered alpha coherence, with shifts in network dynamics underlying the observed cognitive effects.

Viewed from the large-scale networks’ perspective, our findings only partially concur with the literature discussed above. Instead of previously reported stronger coupling between hubs of the DMN (i.e., ACC) and hubs of the ECN (i.e., pars orbitalis as part of the IFG; Beaty et al., 2016), we found a tRNS-induced decrease in alpha coherence change between the SN (i.e., ACC) and ECN (pars orbitalis as part of the IFG). Similarly, a tRNS-induced decrease in alpha coherence change was observed between the PCC and the medial orbitofrontal cortex. Given that the medial orbitofrontal cortex is part of the medial frontal cortex (i.e., of the DMN), our results may be indicative of a tRNS-induced reduction of alpha coherence within the DMN itself. This decreased DMN activity is in line with the proposed two-stage process model of creativity (Basadur et al., 1982; Ellamil et al., 2012) according to which the ECN is active in the evaluative, last stage of the creative process, whereas the DMN is active only in the generative first stage. While this study could not confirm the previously reported increased coupling between the DMN and ECN core nodes, it indicates the need for creativity research to transition to the large-scale network level and emphasizes the necessity of accounting for the intricate interplay of different brain regions involved in creative processes.

The involvement of the precuneus in the DMN remains a subject of debate. While some studies suggest its involvement with the DMN due to its increased activity during rest (Ellamil et al., 2012; Utevsky et al., 2014), other studies point to its limited role in tasks requiring internal awareness and inhibition of sensory stimulation (typically attributed to the DMN function), such as idea generation, and highlight its property of gathering information from the environment (a property that is untypical for the DMN function; Fink et al., 2010, 2012). Indeed, the activity of precuneus increased with the increase of task constraints, for example, in tasks requiring gathering stimulus-related information (Fink et al., 2012). The observed increase in coherence of the right precuneus with the right paracentral regions, the right inferior temporal lobe, and the left transverse temporal lobe as found in the current study may be indicative of an enhanced coupling within the DMN nodes. Yet, such an increase in activity of the DMN would be expected during the idea generation phase rather than toward the final phase of the creative process.

In contrast to previous studies in which EEG was recorded during task execution (e.g., Ellamil et al., 2012; Fink et al., 2018, 2019; Rominger et al., 2019), we recorded resting-state EEG prior and after the application of tRNS and execution of two verbal DT tasks. While this design precludes a direct comparison with previous investigations measuring EEG during task performance, the concurrent application of tRNS and the execution of DT verbal creativity tasks as employed in our study provides insights into the intricate neurophysiological mechanisms underlying tRNS effects within the realm of creativity.

### Frontal Cortex and Idea Evaluation

The DLPFC, as part of the frontal cortex and the stimulation target in the current study, is an important working memory hub (Fregni et al., 2005; Mottaghy et al., 2000) that is also linked to a selection of task-relevant and a suppression of task-inappropriate thoughts (Bunge et al., 2001; Metzuyanim-Gorlick & Mashal, 2016). By effective guiding of thoughts toward a specific goal and evaluating the suitability of potential solutions with respect to context and task demands, idea selection constitutes optimal solution finding (Weinberger et al., 2017). The exploratory analyses examining the effect of tRNS on imaginary coherence between the 62 scouts as defined by the anatomical Mindboggle Atlas (Klein et al., 2017) revealed changes in alpha coherence. While we did not find a significant change in alpha coherence for either the left nor the right frontal regions (see Supporting Information S2.1 and SF1), the change in alpha coherence (specifically wND) was significantly predicted by the tRNS-induced AUT and AFT originality changes for the left frontal areas, and by the AUT originality and flexibility changes for the right frontal areas. Specifically, the regression analysis suggested a suppression effect between changes in AUT originality and flexibility in predicting changes in right frontal alpha coherence. While originality alone indicated a marginal negative effect on alpha coherence and flexibility alone revealed no significant effect, the combination of both revealed a suppression effect. As such, flexibility appears to refine the negative effect of originality on right frontal alpha coherence, enhancing the overall explanatory power of the model. This suggests that the interplay between these cognitive processes contributes more significantly to changes in brain activity than when considered independently.

Considering the current design in which we acquired resting-state EEG shortly before and immediately after the 20-min application of tRNS/sham to the DLPFC, it is likely that the measured coherence changes only captured the brain’s activity of the final creative phase, that is, idea selection. Since the final stage of the creative process contributes to the originality of the answers given (Weinberger et al., 2017), the reported predictive power of originality changes on wND coherence changes of frontal areas, including the DLPFC, seems plausible. In that sense, the results of the current study indicate a link between the behavioral and neurophysiological effects of tRNS. Specifically, the tRNS-induced wND coherence changes in the left frontal cortex, which were predicted by the originality change scores, concur with previous findings of a link between left prefrontal regions and the increased originality and appropriateness of ideas (Benedek et al., 2014; Rominger et al., 2019), further emphasizing the relevance of executive processes in overcoming dominant but uncreative responses. Indeed, left IFG as a node of verbal creativity selectively activates remote conceptual networks and inhibits related semantic information (Heilman et al., 2003). Also, an increased size of the left IFG was found in individuals performing better in verbal creativity tasks (Zhu et al., 2013) and several fMRI studies reported increased activity of the IFG for more semantically distant answers or inventive concepts (e.g., Green et al., 2010; H. Zhang et al., 2014). Adding to this evidence, the change in coherence that was predicted by the tRNS-induced AUT and AFT originality changes for the left frontal areas concur with a recent meta-analysis on the effects of tDCS on creative thinking, concluding that creative performance in both divergent and CT tasks can be enhanced with anodal tDCS over the left DLPFC (Chen et al., 2024).

While both the AUT and the AFT can be used as measures of creative cognition, they differ with respect to their fundamental cognitive mechanisms. AUT, as a classical DT task, emphasizes idea generation by asking to provide a variety of uses for a given object. Not only does this task require mental imagery, but, more importantly, it requires inhibition of conventional uses to produce more creative responses. Conversely, the AFT is an associative thinking task that assesses, on one hand, the ability to produce responses related to a given set of words and, on the other hand, the organization of associative pathways in those responses. Thus, while the AUT examines creativity through the diversity and originality of ideas, the AFT calls upon associative processes and novelty of connections that contribute to creative thinking. Given these differences in task demands, a stronger association between the activity of the DLPFCs and the AUT as compared with AFT is not surprising.

The lack of clear behavioral effects (i.e., of all the six measures, only AUT originality indicated a significant poststimulation decrease; see Supporting Information S2.2 and SF.2) complicates the direct interpretation of the results. As such, the results of the regression analyses indicate associations but do not allow for inferences about causality. Consequently, the behavioral measures that contributed to the explained variance in the tRNS-induced changes in alpha coherence of the DLPFCs indicate an associative rather than a direct causal relationship. An inclusion of a control task (e.g., a task posing attentional demands without the involvement of DT) would have allowed for a better elucidation of the specificity of these tRNS-induced alpha coherence changes and their relation to creativity.

The choice of the statistical infrequency approach to assess originality in a sample of 40 participants comes with several challenges (Forthmann et al., 2020). Small samples limit the variability of ideas, leading to less reliable and more unstable originality scores. Since in the statistical infrequency approach originality rating is based on how frequently an idea occurs, rare responses may be overestimated, inflating originality scores that might not hold in larger samples. With fewer participants, randomness plays a bigger role, deeming it harder to distinguish truly creative ideas from those that are simply uncommon by chance. Overall, samples below 200 reduce the precision of infrequency-based originality estimates that may potentially lead to misleading conclusions, where the findings might not generalize to larger populations. Nevertheless, the statistical infrequency approach as applied in this study ensures that the findings are based solely on objective, quantitative measures, thereby minimizing the potential for subjective biases. Additionally, this approach offers a more time- and cost-effective method of assessing originality, as it avoids the need for human raters and allows for a streamlined analysis process. Despite its limitations (Forthmann et al., 2020), the choice of this objective method to assess participants’ originality aligns with the overarching goal of establishing reproducible and efficient assessments of creative performance (Camenzind et al., 2024; Gray et al., 2019; Johnson et al., 2021).

The present study provides insights into the neural mechanisms underlying creativity and the role of tRNS in modulating brain activity associated with idea selection and evaluation. By demonstrating that tRNS-induced changes in wND coherence of the DLPFC are predicted by changes in originality and flexibility, this research underscores the DLPFC’s role in executive processes that foster creative thinking. Moreover, these findings contribute to a growing body of literature that highlights the importance of the left prefrontal cortex in generating original and appropriate ideas, thus offering a neurophysiological basis for enhancing creativity through noninvasive brain stimulation techniques. Future research could explore the long-term effects of tRNS on creative cognition and extend these findings to different creativity tasks. Investigating the interplay between other brain regions involved in creativity could further elucidate the comprehensive neural networks that support creative thinking.

## Supporting Information

Supporting information for this article is available at https://doi.org/10.1162/netn_a_00446.

## Author Contributions

Magdalena Camenzind: Conceptualization; Data curation; Formal analysis; Investigation; Visualization; Writing – original draft; Writing – review & editing. Rahel A. Steuri: Data curation; Investigation; Writing – review & editing. Branislav Savic: Investigation; Writing – review & editing. Fred W. Mast: Funding acquisition; Writing – review & editing. René M. Müri: Conceptualization; Funding acquisition; Visualisation; Writing – review & editing. Aleksandra K. Eberhard-Moscicka: Conceptualization; Investigation; Project administration; Supervision; Visualization; Writing – original draft; Writing – review & editing.

## Funding Information

René M. Müri, Schweizerischer Nationalfonds zur Förderung der Wissenschaftlichen Forschung (https://dx.doi.org/10.13039/501100001711), Award ID: 175615. René M. Müri and Fred W. Mast, Interfaculty Research Cooperation (IRC) “Decoding Sleep” of the University of Bern.

## Data Deposition

Data and connectivity analysis codes are available at the following URL: https://osf.io/r67fj/.

## Supplementary Material



## TECHNICAL TERMS

Divergent thinking (DT): Typically required when there is more than one correct solution due to vague selection criteria.
Convergent thinking (CT): Typically required for persistence and focus to find one single solution to well-defined problems.
Executive control network (ECN): Primarily involved in the execution of decisions and idea selection.
Default mode network (DMN): Activates during inward-focused, memory-based learning.
Weighted node degree (wND): Graph theoretical measure corresponding to the strength of connections between a given node and all the other nodes.
Minimum-norm imaging (MN imaging): Estimates amplitudes of brain sources by means of noise statistics that are specified by the noise covariance matrix.
Dynamical Statistical Parametric Mapping (dSPM): Scales current density maps with noise covariance yielding standardized z-scores.
Vertex: In the Brainstorm toolbox, location of a node within a grid used for EEG source localization.
Imaginary coherence (IC): Measure of functional connectivity that quantifies the time-lagged synchronization between signals, hence avoiding spurious interactions due to volume conduction.
Scout: In the Brainstorm toolbox, a scout refers to a “region of interest.”
Transcranial Random Noise Stimulation (tRNS): Uses alternating current with random amplitudes and frequencies (typically between 100 and 700 Hz) to increase neuronal sensitivity.
