# Supplementary material for: The impact of transcranial random noise stimulation (tRNS) on alpha coherence and verbal divergent thinking
Source: Netw Neurosci. 2025 Apr 30;9(2):569–90. doi: 10.1162/netn_a_00446 (PMC12140578; doi:10.1162/netn_a_00446)
Supplement: Supplementary file 1 [file netn-9-2-569-s001.pdf]

Supplementary Material

S1. Additional behavioural measures collected during the screening and the two experimental sessions

S1.1 Inventory of Creative Activities and Achievements (ICAA)

The Inventory of Creative Activities and Achievements (ICAA, Diedrich et al., 2018) is a self-report instrument designed to assess the frequency of engagement in everyday creative activities and the level of creative achievements across eight creative domains (e.g., literature, music, crafts). The ICAA comprises two scales. The first scale measures creative achievement (CAch), distinguishing eleven different levels of achievement in each domain. For this study, the scale for creative activities (CAct) was utilized, which queries the frequency of specific activities over the past ten years on a 5-point Likert scale (0 = "never"; 1 = "1-2 times"; 2 = "3-5 times"; 3 = "6-10 times"; 4 = "more than 10 times"). Each domain comprises six items. To determine a domain score, the mean of the responses was calculated, and the total score was obtained by summing the average values across all the eight domains.

S1.2 Abbreviated Torrance Test for Adults (ATTA)

The ATTA evaluates verbal and figural creative thinking. It comprises of three subtasks: one verbal and two figural. In the initial verbal subtask, participants are prompted to list potential consequences of being able to walk on air or fly without an aircraft. The second subtask involves completing and titling two incomplete figures. In the last subtask, participants are presented with nine triangles, and they are instructed to use them as a foundation for generating multiple drawings, each with a unique title. Every subtask is timed for three minutes. All responses were evaluated according to the scoring manual and resulted in a final total score (Goff & Torrance, 2022).

S1.3 Mehrfachwahl-Wortschatz-Intelligenztest (MWT)

Verbal intelligence was estimated by means of the Mehrfachwahl-Wortschatz-Intelligenztest (MWT, Lehrl et al., 1995). In this test, participants were presented with 37 sets of letter-strings, whereby each set consisted of three pseudowords and one real word, which had to be identified. The final score was determined by the number of correctly identified words.

S1.4 Wiener Matrizentest (WMT-2)

Nonverbal intelligence was evaluated using the Wiener Matrizentest (WMT-2) under conditions devoid of time constraints (Formann et al., 2011). Each of the 18 experimental items comprise a three-by-three matrix, with the final square left empty. The matrix is systematically organized, adhering to predefined rules for each square. Participants were presented with eight further matrices for completing the last, vacant square based on the observed pattern, and were tasked with selecting the most fitting pattern. The final score comprised of the number of correct responses.

S1.5 Stroop Test

A variant of the Victoria Stroop Test (Spreen & Strauss, 1998) was utilized to evaluate inhibitory control. Participants were instructed to name the colour of dots or words across three subtasks, each differing in interference level. In the first subtask, participants named the colours of dots. In the second subtask, they identified the colour of a word that did not match its semantic meaning (e.g., the word "and" written in red), while in the last subtask they had to name the colour of a word that was incongruent with the colour of its letters (e.g., the word "red" written in green). The final stroop score was derived by subtracting the time taken to complete the first subtask from that required for completion of the last subtask.

S1.6 NEO-Five Factor Inventory (NEO-FFI)

The German version of the short personality inventory (NEO Five-Factor Inventory, NEO-FFI, Borkenau & Ostendorf, 2008) was used in this study. NEO-FFI is structured around five personality dimensions (i.e., Neuroticism, Extraversion, Conscientiousness, Agreeableness, and Openness to Experience), with 12 items corresponding to each of the five personality dimensions that were evaluated according to the inventory's manual.

S1.7 Sleep Questionnaire (SF-A/R)

The German Sleep Questionnaire SF-B/R (Görtelmeyer, 2011) facilitates both quantitative and qualitative analysis of sleep behaviour and experiences. Comprising 25 questions, the questionnaire pertains to the preceding night's sleep. In the current study participants were instructed to complete the questionnaire in the morning of the day of each of the three study sessions. This questionnaire serves to ascertain various sleep indices, such as difficulty falling asleep (EES), difficulty maintaining sleep (DSS), early awakening (VZA), overall sleep characterization (ASC), and total sleep duration. It also assesses sleep quality (SQ), post-sleep recovery (GES), pre-sleep psychological balance (PSYA), pre-sleep psychological fatigue (PSYE), and psychosomatic symptoms during sleep (PSS). Evaluation of the questionnaire was conducted in accordance with the manual.

S1.8 Positive Negative Affect Scale (PANAS)

The Positive Negative Affect Scale (PANAS, Watson, et al., 1988), comprises of two subscales and includes items related to positive (PA) and negative affect (NA). The PA subscale measures feelings of interest, excitement, strength, enthusiasm, pride, vigour, inspiration, determination, attentiveness, and activity. Conversely, the NA subscale evaluates sensations of stress, upset, guilt, fear, hostility, irritability, shame, nervousness, jitteriness, and fearfulness. Each of the 20 items is rated on a scale ranging from "very slightly or not at all" to "extremely." Scores for PA items and NA items are tallied separately. Subscale scores range from 10 to 50, with higher scores indicating greater levels of positive or negative affect.

S1.9 Visual Analogue Scale (VAS)

At each study session, participants' alertness was measured by means of a visual analogue scale (VAS). The VAS measures alertness levels on a continuum from "not awake at all" to "completely awake" whereby the values range from 0 to 10 (with higher values indicating higher alertness).

ST1. Behavioural measures collected during the three sessions (i.e., screening session, active tRNS session, and sham tRNS session).

Measures	Screening session M (SD)	tRNS session M (SD)	Sham tRNS session M (SD)
**Inventory of Creative Activities and Achievements (ICAA)**			
total score	18.286 (4.421)	-	-
Literature	1.740 (0.598)	-	-
Music	2.268 (1.069)	-	-
Crafts	3.061 (1.149)	-	-
Cooking	3.044 (1.011)	-	-
Sports	1.927 (0.858)	-	-
Art	2.159 (0.751)	-	-
Performing arts	1.698 (0.621)	-	-
Technical	2.390 (1.079)	-	-
**Abbreviated Torrance Test for Adults (ATTA)**		-	-
total score	61.927 (4.421)		
**Mehrfachwahl-Wortschatz-Intelligenztest (MWT)**		-	-
final score	30.415 (2.072)		
**Wiener Matrizen-Test (WMT-2)**		-	-
final score	12.341 (3.572)		
**Stroop task**		-	-
final score (in sec.)	12.9 (7.0)		
**NEO - Five Factor Inventory (NEO-FFI)**			
Neuroticism	34.122 (3.671)	-	-
Extraversion	37.463 (2.795)	-	-
Openness	34.098 (3.214)	-	-
Agreeableness	34.390 (4.084)	-	-
Conscientiousness	40.195 (2.549)	-	-
**Sleep-Questionnaire (SF-A/R)**			
Difficulty falling asleep (score)	2.95 (1.244)	3.35 (1.013)	3.175 (1.070)
Difficulty sleeping through the night (score)	2.112 (1.217)	2.250 (1.198)	2.225 (1.072)
Waking up prematurely (score)	1.500 (1.323)	1.600 (1.428)	1.400 (1.200)
General sleep characterization (score)	3.979 (0.723)	4.071 (0.783)	4.090 (0.650)
Total Sleep Time (h)	7.466 (2.617)	7.760 (1.820)	7.760 (0.900)
Sleep Quality (score)	1.865 (0.594)	1.682 (0.506)	1.778 (0.547)
Feeling of being refreshed after sleep (score)	3.304 (0.547)	3.279 (0.596)	3.468 (0.555)
Mental balance before going to sleep (score)	14.450 (2.224)	14.875 (2.648)	14.925 (2.240)
Mental exhaustion before going to sleep (score)	3.065 (0.857)	3.080 (0.701)	2.845 (0.694)
Psychosomatic symptoms in the sleep phase (score)	1.130 (0.182)	1.130 (0.203)	1.135 (0.170)
**Positive Negative Affective Schedule (PANAS)**			
positive affect score	2.932 (0.506)	2.987 (0.490)	3.053 (0.519)
negative affect score	1.268 (0.299)	1.325 (0.296)	1.305 (0.287)
**Wakefulness (VAS)**			
score	7.208 (1.520)	6.608 (1.792)	6.928 (1.454)

Note: Mean values (M) and standard deviation (SD) of the measures collected from 40 healthy participants during the three sessions.

ST2. tRNS-related sensations as perceived and reported by 40 healthy participants following the tRNS and sham sessions.

Item	tRNS condition		Sham condition	
	Most reported	N	Most reported	N
Headache	absent	30	absent	33
Neck pain	absent	29	absent	28
Pain on scalp	absent	40	absent	37
Burning	absent	29	absent	31
Itching	weak	20	weak	24
Redness of skin	absent	40	absent	40
Fatigue	weak	15	weak	18
Difficulty concentrating	absent	17	absent	26
Mood changes	absent	26	absent	35
correct guess of stimulation condition	24		24	

Note: While more than half of the study participants correctly guessed the stimulation condition, the relationship between these subjective ratings and the actual stimulation condition (i.e., tRNS or sham) was not significant ($\chi 2 = 2.45$, df = 1, $p = .1175$).

S2. Analysis of behavioural data and neuronal measures collected during the two experimental sessions

S2.1 Analysis of alpha coherence

Two paired t-tests were conducted to compare the pre and post alpha coherence change in the left and right frontal areas between the two conditions (i.e., tRNS and sham) indicating no significant change in the post-pre alpha coherence (both p's > 0.280).

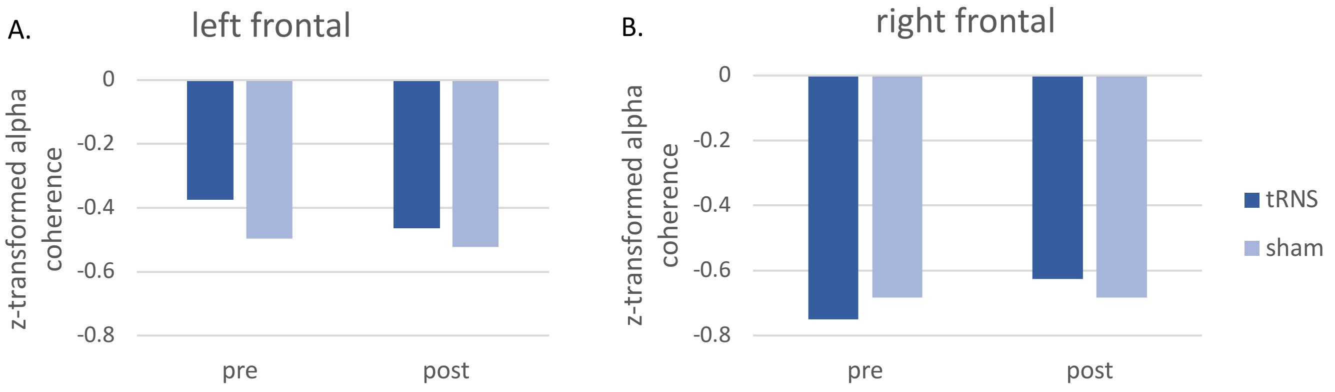

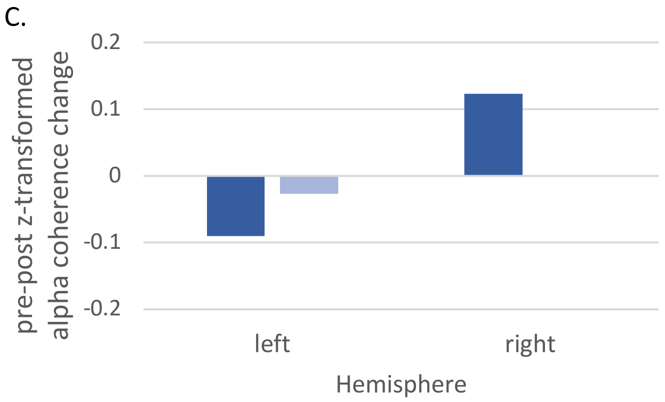

SF1. Z-transformed alpha coherence values for the left and right frontal brain regions under tRNS and sham conditions. A. Bar plots of pre- and post-intervention z-transformed alpha coherence for the left frontal region. Dark blue bars represent the tRNS condition, and light blue bars represent the sham condition. B. Bar plots of pre- and post-intervention z-transformed alpha coherence for the right frontal region. Dark blue bars represent the tRNS condition, and light blue bars represent the sham condition. C. Bar plots of the post-pre change in z-scored alpha coherence for the left (left) and the right (right) frontal regions. Dark blue bars represent the tRNS condition, and light blue bars represent the sham condition.

S2.2 Analysis of behaviour

Behavioural performance in both tasks (i.e., AUT and AFT) was assessed using measures of fluency, flexibility, and originality. Six paired t-tests were conducted to assess the post-tRNS change in behaviour. Of the six paired t-tests, only AUT originality showed a significant reduction indicating a post-tRNS reduction in AUT originality ($t(39) = -3.036$, $p = .004$, see SF1, all other p's > .088).

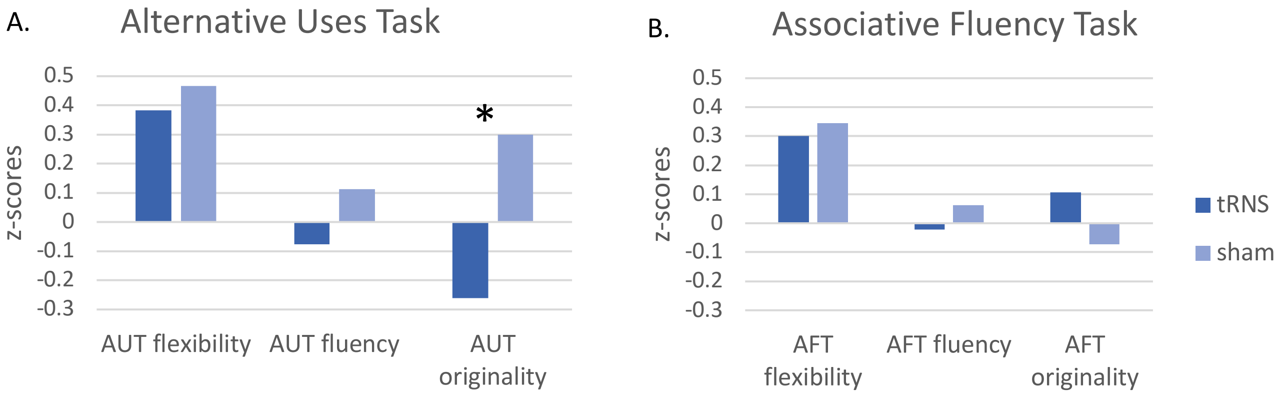

SF2. Z-transformed performance values for fluency, originality, and flexibility for the two experimental conditions, i.e., transcranial Random Noise Stimulation (tRNS) and sham. Panel A. Bar plots of z-transformed scores for the three measures—fluency, originality, and flexibility—in the Alternative Uses Task (AUT). Dark blue bars represent the tRNS condition, and light blue bars represent the sham condition. B. Bar plots of z-transformed scores for the three measures—fluency, originality, and flexibility—in the Associative Fluency Task (AFT). As in A., dark blue bars represent the tRNS condition, and light blue bars represent the sham condition.

References

Borkenau, P., & Ostendorf, F. (2008). NEO-FFI: NEO-Fünf-Faktoren-Inventar nach Costa und McCrae, Manual.

Diedrich, J., Jauk, E., Silvia, P. J., Gredlein, J. M., Neubauer, A. C., & Benedek, M. (2018). Assessment of real-life creativity: The Inventory of Creative Activities and Achievements (ICAA). *Psychology of Aesthetics, Creativity, and the Arts*, *12*(3), 304.

Formann, A. K., Waldherr, K., & Piswanger, K. (2011). *Wiener Matrizen-Test 2: Ein Rasch-skalierter sprachfreier Kurztest zu Erfassung der Intelligenz; Testheft*. Hogrefe.

Goff, K., & Torrance, P. E. (2022). Abbreviated Torrance Test for Adults: Manual. *Scholastic Testing Service*.

Görtelmeyer, R. (2011). *SF-A/R und SF-B/R: Schlaffragebogen A und B*.

Lehrl, S., Triebig, G., & Fischer, B. (1995). Multiple choice vocabulary test MWT as a valid and short test to estimate premorbid intelligence. *Acta Neurologica Scandinavica*, *91*(5), 335–345.

Spreen, O., & Strauss, E. (1998). A compendium of neuropsychological tests: Oxford University Press. *New York*, 213–218.

Watson, D., Clark, L. A., & Tellegen, A. (1988). Development and validation of brief measures of positive and negative affect: the PANAS scales. *Journal of Personality and Social Psychology*, *54*(6), 1063.
